# A Literature Review of Social and Economic Leader–Member Exchange

**DOI:** 10.3389/fpsyg.2020.01474

**Published:** 2020-07-08

**Authors:** Ingvild Andersen, Robert Buch, Bård Kuvaas

**Affiliations:** ^1^Department of Leadership and Organizational Behavior, BI Norwegian Business School, Oslo, Norway; ^2^School of Communication, Leadership and Marketing, Kristiania University College, Oslo, Norway

**Keywords:** leader–member exchange, social exchange, economic exchange, literature review, leader–follower relationships

## Abstract

Leader–member exchange (LMX) research has increasingly relied upon the social exchange theory (SET) as a theoretical foundation, but the dominating way of measuring LMX has not followed this theoretical development (Gottfredson et al., 2020). With the aim of developing a measure that more coherently reflects SET, Kuvaas et al. (2012) conceptualized LMX as two qualitatively different relationships, labeled economic LMX and social LMX. Since the most applied LMX measures are under scrutiny for not being sufficiently grounded in theory (Gottfredson et al., 2020), it may be especially important to expose alternative measures. Therefore, we provide a comprehensive review of the research to date applying a two-dimensional approach to LMX, while also adding to interpretation and suggestions for how we can progress the field even further.

## Introduction

The leader–member exchange (LMX) literature is hardly at its infancy, but the field is still under progressive development (Martin et al., 2019). It seems as if the LMX field is reinventing itself, as indicated by its entrance in novel terrains (e.g., social network analysis; Sparrowe and Emery, 2015); its application of more sophisticated methods (e.g., polynomial regression, multilevel modeling; Kim et al., 2019); and its update of theoretical influences (e.g., Thomas et al., 2013; Sparrowe, 2018). Nevertheless, a central concern in the LMX literature’s development has been the emphasis on the need for more coherence between theory and empirical research (Krasikova and LeBreton, 2012; Gottfredson et al., 2020). One particular important issue that has been raised is that most of the extant measures of LMX do not sufficiently reflect its theoretical foundation (Bernerth et al., 2007; Gottfredson et al., 2020). In what follows, we aim to contribute to LMX literature by providing a specialized review of a growing stream of research that adopts an alternative LMX conceptualization and measurement that are more strongly anchored to its contemporary theoretical foundation.

The LMX theory revolves around the notion that leaders often interact differently with various followers (Graen and Uhl-Bien, 1995). With some followers, there may be a great deal of personal involvement, trust, and long-term investment. While with others, there may be less investment and trust, and more formal, *quid pro quo* transactions. Both of these types of exchange relationships are theoretical underpinnings of the social exchange theory (SET) (Blau, 1964), in which the LMX theory has increasingly relied on as a theoretical framework (e.g., Dulebohn et al., 2012; Matta and Van Dyne, 2015; Gottfredson et al., 2020). In addition, whereas role theory, which was the original theoretical underpinnings to LMX (e.g., Graen, 1976), failed to receive meta-analytical support, the SET strongly did so (Martin et al., 2016). Still, measures of the LMX relationship have traditionally focused on the first, meaning the “socioemotional” exchange relationship (e.g., Bernerth et al., 2007; Wayne et al., 2009), as LMX has typically been measured along a low- to high-quality continuum, where the items are purely targeted at capturing socioemotional qualities. Therefore, lower levels of LMX merely reflect the absence of features that are characteristic of a high-quality LMX relationship, and not an economic, contractual *quid pro quo* relationship. Although prior research has contributed to important insights regarding the benefits of high-quality LMX relationships (e.g., Gottfredson and Aguinis, 2017), it has neglected to capture the role of economic qualities of LMX relationships. This aligns well with Gottfredson et al. (2020) critique that most of the extant LMX measures “…do not capture LMX’s theoretical foundations” and that there is “misalignment between conceptualization and measurement” (p. 1).

In order to sufficiently capture both the economic and socioemotional exchange relationships, Kuvaas et al. (2012) conceptualized LMX as two qualitatively different relationships, labeled economic and social LMX. In alignment with the SET, social LMX relationships are characterized by a high degree of trust and long-term investment, which generates diffuse obligations, a sense of being taken care of by the other, and an anticipated mutuality in exchanges (Shore et al., 2006). An economic LMX relationship, on the other hand, is more formal and instrumental, and there is less interpersonal trust that the other will reciprocate future obligations, making exchanges between them more *quid pro quo* (Kuvaas et al., 2012). This conceptualization aligns strongly with the contemporary and dominating theory explaining LMX relationships, namely the SET, as recently called for by Gottfredson et al. (2020) in their critique of prior LMX research.

Prior larger reviews and meta-analyses have focused on the importance of high-quality LMX relationships (e.g., Gerstner and Day, 1997; Dulebohn et al., 2012) and have offered valuable insights on the benefits of being in a high-quality LMX relationship. However, they provide limited insight regarding the economic type of relationship between a leader and a follower. Therefore, we review research that has taken a two dimensional approach to LMX with the aim of exploring its relevance and importance for LMX research overall (for full overview of included articles see Table 1). As such, our review is the first to provide a coherent overview of the specific implications offered by adopting a two-dimensional approach to LMX. As discussed throughout our paper, LMX relationships do exhibit both socioemotional and economic aspects. Usually, social and economic LMX are moderately negatively related to each other (e.g., Berg et al., 2017), but research also suggests that they may interact and that a relationship may carry high levels of both (Caniëls and Hatak, 2019). In addition, economic LMX seems to provide unique explanatory variance on a range of various employee outcomes beyond what a high-quality or social LMX can do by its solitary (e.g., Kuvaas et al., 2012; Buch et al., 2019a) (see Table 2 for economic LMX correlations). This indicates that the two-dimensional approach to LMX has allowed to more fully capture the nature of the LMX relationship, and overall contributed to an enhanced understanding of the leader–follower relationship. Therefore, we argue that this line of research warranted special attention as offered throughout our review.

**TABLE 1:** Overview of published studies applying the two-dimensional social and economic LMX.

Article	Journal	ELMX correlations	SLMX correlations
Aleksić et al. (2017)	Personnel Review	Non-significant relationship with creativity; satisfaction with work–family balance; LMX7 and SLMX.	Positive relationship with creativity; satisfaction with work–family balance and LMX7.
Babic et al. (2019)	Journal of Knowledge Management	Non-significant relationship with prosocial motivation; knowledge hiding and SLMX.	Positive relationship with prosocial motivation. Non-significant relationship with knowledge hiding.
Berg et al. (2017)	European Management Journal	Non-significant relationship with creative behavior and willingness to take risks. Negative relationship with emotional carrying capacity and SLMX.	Positive relationship with creative behavior and emotional carrying capacity. Non-significant relationship with willingness to take risks.
Buch (2015)	International Journal of Human Resource Management	Negative relationship with intrinsic motivation; affective commitment and SLMX.	Positive relationship with intrinsic motivation and affective commitment.
Buch et al. (2019a)	Journal of Organizational Behavior	Negative relationship with affective commitment; other orientation; OCB; work effort and SLMX. Positive relationship with turnover intention.	Positive relationship with affective commitment; other orientation; OCB and work effort. Negative relationship with turnover intention.
Buch et al. (2014a)	Leadership and Organizational Development Journal	Positive relationship with extrinsic motivation. Negative relationship with leader rated work effort and SLMX. Non-significant relationship with intrinsic motivation.	Positive relationship with intrinsic motivation and leader rated work effort. Non-significant relationship with extrinsic motivation.
Buch et al. (2014b)	Journal of Leadership and Organizational Studies	Positive relationship with laissez-faire leadership. Negative relationship with affective commitment; work effort; OCB and SLMX.	Negative relationship with laissez-faire leadership. Positive relationship with affective commitment; work effort and OCB.
Caniëls and Hatak (2019)	The International Journal of Human Resource Management	Positive relationship with narcissism. Non-significant relationship with employee resilience and SLMX.	Non-significant relationship with narcissism. Positive relationship with employee resilience.
Dysvik et al. (2015)	Leadership and Organization Development Journal	Non-significant relationship with employee knowledge donating and manager knowledge collecting. Negative relationship with SLMX.	Positive relationship with employee knowledge donating. Non-significant relationship with manager knowledge collecting.
Kuvaas and Buch (2018)	Human Resource Management	Positive relationship with perceived invariable goals. Negative relationship with leader rated work performance and SLMX.	Negative relationship with perceived invariable goals. Positive relationship with leader rated work performance.
Kuvaas and Buch (2020)	Leadership and Organization Development Journal	Negative relationship with need for relatedness; need for autonomy; need for competence and SLMX. Positive relationship with turnover intention. Non-significant relationship with leader self-efficacy and leader role ambiguity.	Negative relationship with leader role ambiguity and turnover intention. Positive relationship with need for relatedness; need for autonomy and need for competence. Non-significant relationship with leader self-efficacy.
Kuvaas et al. (2012)	The Leadership Quarterly	Negative relationship with manager rated work performance; manager rated OCB and SLMX.	Positive relationship with manager rated work performance and manager rated OCB.

*SLMX, Social leader–member exchange; ELMX, Economic leader–member exchange; OCB, Organizational citizenship behavior.*

**TABLE 2:** Overview of economic LMX correlations or range of correlations from published studies.

Variable	ELMX correlations
SLMX	*r* = −0.15**, −0.49**
Affective commitment	*r* = −0.34**, −0.22**
Turnover intention	*r* = 0.20**, 0.23**
Satisfaction with work–family balance	Non-significant
Perceived invariable goals	*r* = 0.36**
Knowledge hiding	Non-significant
Employee knowledge donating	Non-significant
Intrinsic motivation	*r* = −0.33**
Extrinsic motivation	*r* = 0.15**
Prosocial motivation	Non-significant
Need for relatedness	*r* = −0.19**
Need for competence	*r* = −0.19**
Need for autonomy	*r* = −0.26**
OCB	*r* = −0.19**, −0.12**
Manager rated OCB	*r* = −0.24**
Work effort	*r* = −0.28**, −0.14**
Leader rated work effort	*r* = −0.14**
Manager rated work performance	*r* = −0.29**, −0.25**
Creativity	Non-significant
Other orientation	*r* = −0.06**
Emotional carrying capacity	*r* = −0.16*
Willingness to take risks	Non-significant
Narcissism	*r* = 0.22*
Employee resilience	Non-significant
Laissez-faire leadership	*r* = 0.31**, 0.33**
Manager knowledge collecting	Non-significant
Leader self-efficacy	Non-significant
Leader role ambiguity	Non-significant

SLMX, Social leader–member exchange; ELMX, Economic leader–member exchange; OCB, Organizational citizenship behavior. *p < 0.05. **p < 0.01.

## Theoretical Underpinnings of Social and Economic LMX

Although the dominating view today seems to be that LMX strongly relies on the SET (e.g., Bernerth et al., 2007; Colquitt et al., 2014), this has not always been the case. Actually, when introduced to the scholarly literature close to 50 years ago, the LMX theory was referred to as the vertical dyad linkage (VDL) theory, and its main focus was understanding how a “role-making” process can occur naturally (Dienesch and Liden, 1986; Graen and Scandura, 1987). After some time, the VDL theory, which started out as an alternative to the notion that leaders treat everyone the same (“average leadership style”; cf. Graen and Uhl-Bien, 1995), was “rebranded” to the “LMX theory” and started increasingly to rely on Blau (1964) SET and the distinctions between a social and an economic exchange relationship.

In a similar fashion, in the related area of employee–organization relationships (EOR), several constructs have been used as proxy indicators for the nature or quality of a particular social exchange relationship (Takeuchi, 2012) – including perceived organizational support (POS; Dulac et al., 2008; Liao, 2011), the psychological contract (e.g., Van Dyne and Ang, 1998), perceived investment in employee development (e.g., Lee and Bruvold, 2003), justice (Masterson, 2001), commitment (e.g., Walumbwa et al., 2011), and perceived supervisor support (Settoon and Mossholder, 2002). Still, until Shore et al. (2006) developed measures of both social and economic exchange relationships with the organization, most research on the EOR had overlooked the role of economic exchanges, as has been the case for LMX research.

According to the SET, perceived social exchange relationship is characterized by a long-term orientation, since the exchange is ongoing and based on feelings of diffuse obligation. The emphasis is on socioemotional aspects of exchange, such as give and take and being taken care of, and each exchange partner trusts that the other will reciprocate (Shore et al., 2006). Social exchange entails a broad investment, as it involves both the exchange of socioemotional resources as well as investment in the relationship itself (Shore et al., 2009). These characteristics allow for transitory perceived asymmetries between contributions and inducements because one party is able to prioritize the other party’s interests ahead of their own (Deckop et al., 1999).

Employees with a strong perception of social exchange will thus be prosocially motivated to a greater degree – that is, they will feel a greater obligation to reciprocate the benefits and support received by engaging in behaviors that exceed the minimum requirements for employment. As was enumerated by Blau (1964) and has been echoed by Foa and Foa (1975) and Shore et al. (2006), the nature of a particular exchange relationship is defined by a person’s interpretation of the meaning of a particular exchange. Increased organizational investment, for instance, should create feelings of diffuse obligations on behalf of the employees, which, in turn, should influence employees to increase their efforts above and beyond the minimum requirements (Shore et al., 2006) – not solely to garner future benefits but also as an expression of appreciation (Blau, 1964). Shore et al. (2006) and Song et al. (2009) provide support for this proposition by demonstrating that social exchange perceptions are associated with both improved work performance and organizational citizenship behaviors (OCBs).

Unlike social exchange, employees with a strong economic exchange perception probably see their relationship with their leader (and the organization as a whole; see Loi et al., 2009) as involving a set of financial and tangible obligations in exchange for the fulfillment of job duties (Shore et al., 2009). An economic exchange relationship is not expected to be long term (Shore et al., 2006). It is characterized by little personal involvement (Lai et al., 2009) and involves the exchange of more financial or tangible resources (Song et al., 2009), typically obtained via discrete *quid pro quo* transactions (Lai et al., 2009). As a result, most contemporary research on the EOR differentiates social from economic exchanges on the following dimensions: the level of investment and obligation, the degree of trust, the immediacy of the exchange, and the financial vs. socioemotional aspect of the exchange (Shore et al., 2006, 2009).

## Measurement of Social and Economic LMX Relationships

With respect to measuring LMX, the most frequently utilized indicators (e.g., LMX7) have been criticized for lacking content validity (e.g., Bernerth et al., 2007; Colquitt et al., 2014; Gottfredson et al., 2020). Furthermore, Sparrowe and Liden (1997, p. 524) noted that applying the SET to LMX research has been problematic because “the dimensions of actual exchange behavior that differentiate economic from social exchange have not been specified in a way that facilitates empirical verification.”

Indeed, not only Gottfredson et al. (2020) but also Colquitt et al. (2014) advocated the use of alternative scales that are more connected to the sentiments that Blau (1964) used to describe exchange relationships. In their comparison of the relative content validity of scale indicators of social exchange relationships, Colquitt et al. (2014) found a supervisor targeted version of Shore et al. (2006) social exchange scale (a social exchange-based measure of leader–member relationships; i.e., LMX) to exhibit a content-valid pattern. In line with this later criticism, reiterated by Gottfredson et al. (2020) and Kuvaas et al. (2012) did, in fact, base their first measures of social and economic LMX simply by using Shore et al. (2006) measure of perceived social and economic exchange relationships with organizations. As such, social LMX, also sometimes referred to as SLMX, is simply a more content valid supervisor-targeted version of Shore et al. (2006) social exchange scale. As stated by Kuvaas et al. (2012), for most items, they merely replaced “my organization” with “my store manager,” and a sample item of social LMX is “I don’t mind working hard today – I know I will eventually be rewarded by my store manager.”

Accordingly, Kuvaas et al. (2012) main contribution was introducing the economic LMX part into the LMX literature. At this point, an interesting parallel can be drawn with Burns (1978) initial proposition of a “transactional–transformational” leadership continuum, which was later contested by Bass (1985), who suggested that the two leadership styles should be viewed as two separate dimensions, rather than as opposite poles of a single continuum. Still, economic LMX is not merely transactional leadership as it takes a *relationship*-based approach to examining the leader–member dyad, rather than merely measuring transactional leader *behaviors*.

Kuvaas et al. (2012) noted that simply changing the referent of the economic exchange items was not enough, in particular because as leaders, line and/or middle managers probably have limited discretion with respect to pay and compensation issues. They, therefore, rewrote items that could be interpreted mainly as pay decisions to issues of formal authority, in line with descriptions of economic or transactional LMX relationships, as “…based on compliance with job descriptions” (Wayne et al., 2009, p. 254) involving formal role-defined relations and unidirectional downward influence (Graen and Uhl-Bien, 1995). Such relationships are posited “…not to evolve beyond what is specified in the employment contract” (Wayne et al., 2009, p. 254) and as limited to the fulfillment of contractual obligations (e.g., Graen and Uhl-Bien, 1995; Wayne et al., 2009; Walumbwa et al., 2011).

Later, several studies picked up this idea and used the economic LMX measure presented by Kuvaas et al. (2012) in line with their calls for additional studies from other businesses and other countries to learn more about the generalizability of their findings. These social and economic LMX scales have been found to have satisfactory psychometric properties in several languages and countries, including the Netherlands (De Ruiter et al., 2016; Caniëls and Hatak, 2019), Norway (Kuvaas et al., 2012; Buch et al., 2014a; Berg et al., 2017), Slovenia (Aleksić et al., 2017; Babic et al., 2019; Premru, 2019), Belgium (Audenaert et al., 2017), and Oman (a small country in the Middle East) (Alkathiri, 2016).

Furthermore, in support of their two-dimensionality and the added value of measuring economic LMX in addition to traditional measures of social exchange with the supervisor (i.e., LMX7; LMSX; LMX-MDM), these studies have shown social and economic LMX to be differentially related to measures such as narcissism, creative behavior, and prosocial motivation (Aleksić et al., 2017; Berg et al., 2017; Babic et al., 2019; Caniëls and Hatak, 2019). Indeed, Aleksić et al. (2017) empirically investigated both social LMX together with LMX7 and found economic LMX to be non-significantly related to both LMX7 and social LMX [the supervisor-targeted social exchange version of Shore et al.’s (2006)]. Besides, Buch et al.’s (2019a) provided additional evidence of the social and economic LMX distinction via the simple observation that other orientation interacted in a different way with economic LMX than with social LMX in two independent samples.

Findings such as these imply that assuming the existence of economic exchanges between leaders and followers from low scores on scales measuring the social exchange part of LMX is not warranted. Accordingly, both theoretically and empirically, low ratings from leaders and followers when using, for instance, LMX7 do not necessarily imply a transactional economic exchange relationship between the two – it could be an indication of laissez-faire leadership or other unknown factors.

However, it should be emphasized that Kuvaas et al. (2012) excluded some of the more contingent *quid pro quo* items from their economic LMX in their exploratory study in order to achieve a statistically significant chi-square in line, which a “…relatively small group of methodologists” (Crede and Harms, 2019, p. 20) argue “is the single best indicator of model misspecification” (Crede and Harms, 2019, p. 20). Given the aforementioned, as well as the current debate in this regard, future research may consider applying and examining the validity of all eight economic LMX items presented by Kuvaas et al. (2012) rather than merely relying on the four that satisfied the chi-square criteria in their exploratory study.

Nevertheless, in a follow-up study, Buch et al. (2011) developed additional items on the basis of the SET (e.g., Blau, 1964) to better capture the *quid pro quo* aspects of economic LMX relationships. This modified economic LMX scale has, to the best of our knowledge, demonstrated satisfactory psychometric properties in at least five independent samples (Buch et al., 2011, 2014b; Buch, 2015). Since Buch (2015) measured both social and economic LMX, together with Shore et al. (2006) measures of social and economic exchanges with the organization, our review also provides an indication of the measure discriminant validity with respect to each other. Specifically, the supervisor targeted social and economic exchange scales only shared 26% variance or less with each other, adding to the line of research on multifoci perspectives of social exchange (e.g., Rupp and Cropanzano, 2002; Lavelle et al., 2007), demonstrating that followers distinguish between their exchange relationships with their leaders and their exchange relationships with their organization.

Furthermore, Dysvik et al. (2015) refined the wording of a few economic LMX items and used a nine-item scale in their research, which has been used in at least two additional studies (Kuvaas and Buch, 2018; Buch et al., 2019a) with satisfactory validity and reliability in several independent samples amounting to more than 5,000 respondents. The nine-item economic LMX scale used in this research can be found in, for instance, Dysvik et al. (2015).

Finally, in terms of psychometric properties, the majority of the studies included in our review suggest that economic LMX explains unique variance in such outcomes as affective commitment, turnover intention, work performance, and work effort when social LMX is included into the model. The significant amount of unique variance explained by economic LMX not only suggests that economic LMX has its own unique value in prediction but also suggests that economic LMX and social LMX, which align well with traditional conceptualizations of LMX (e.g., LMSX; Bernerth et al., 2007), are two discriminant constructs.

## Literature Overview

In their first study, Kuvaas et al. (2012), with a sample of 552 followers and 78 leaders, obtained support for the two-dimensionality approach to LMX relationships. More specifically, they found that economic LMX was negatively related to leader-rated work performance (α_*1*_ = −0.27, *p* < 0.001) and leader-rated organizational citizenship behavior (OCB) (β_*1*_ = −0.22, *p* < 0.001). On the other hand, social LMX was positively related to work performance (α_*2*_ = 0.20, *p* < 0.001) and OCB (β_*2*_ = 0.26, *p* < 0.001).

Extending the support for the social and economic LMX influence on performance measures, Buch et al. (2014a) found a similar pattern investigating economic and social LMX in relation to leader-rated work effort based on a sample of 352 dyads from the public health sector in Norway. They found that social LMX was positively related to work effort (γ = 0.11, *p* < 0.05), and economic LMX was negatively related to work effort (γ = −0.11, *p* < 0.05). Additionally, they investigated the potential moderating role of work motivation and found that followers who were highly intrinsically motivated seem to be less influenced by the benefits of a social LMX relationship (*b*_high_ = −0.02, *p* > 0.05), whereas followers who were less intrinsically motivated had more to gain from a social LMX relationship (*b*_low_ = 0.24, *p* < 0.01). Such interaction results were not found in relation to economic LMX, but they did find a positive relationship between economic LMX and extrinsic motivation (*r* = 0.15, *p* < 0.01).

Kuvaas and Buch (2018) investigated the association between social and economic LMX and work performance through the mediating role of perceiving goals as invariable, which refers to “the extent to which followers believe that the goals are absolute standards that must be met without exception” (p. 236). Since LMX plays an important role in how followers perceive and respond to HR practices (Bos-Nehles and Audenaert, 2019), they hypothesized that social LMX would decrease and economic LMX would increase the likelihood of perceiving goals invariable. With a sample of 204 followers and 59 leaders, they found that social LMX was negatively (γ = −0.36, *p* < 0.001) and economic LMX positively (γ = −19, *p* < 0.05) associated with perceiving goals as invariable. In addition, social LMX was positively related to leader-rated work performance (*indirect “effect”* = 0.09, *p* < 0.05) through perceiving goals as invariable. No indirect association was found for economic LMX.

In another study, Buch (2015) investigated how social and economic LMX function in conjunction with the social and economic organizational exchange relationship in their relations to affective commitment. Results from a two-wave study of 341 followers illustrated that having a social LMX relationship can dampen the negative association between an economic organization exchange relationship and affective commitment. More specifically, the negative association between economic organization exchange and affective commitment was weaker when social LMX was high (*b*_high_ = −0.22 *p* < 0.01) compared to when social LMX was low (*b*_low_ = −0.41, *p* < 0.001).

As the theoretical argument stands, social and economic LMX relationships exist simultaneously (Goodwin et al., 2009). One study that considered the dual role of social and economic LMX is the one by Caniëls and Hatak (2019). More specifically, they investigated how various *combinations* of social and economic LMX, in conjunction with employee narcissism, influenced employee resilience. In order to do so, they applied polynomial regression on their sample of 123 followers. The results revealed that as long as social LMX dominated over economic LMX, there was a positive association with employee resilience. Still, the results were somewhat different for followers who had narcissistic tendencies. These types of followers benefited the most from a combination of either a low economic and low social LMX relationship or a high economic and high social LMX relationship, indicating that they may respond differently to social and economic LMX.

Taking on another follower characteristic, Buch et al. (2019a) investigated the role of other orientation, in the relationship between social and economic LMX and turnover intention, work effort, affective commitment, and OCB. One sample, which constituted a two-wave study of 200 followers, revealed weaker associations between economic LMX and both turnover intention (*b*_high_ = −0.04, *p* = 0.39 vs. *b*_low_ = 0.38 *p* < 0.01) and affective commitment (*b*_high_ = 0.01 *p* = 0.48 vs. *b*_low_ = −0.35 *p* < 0.01) for followers with higher other orientation. Similar patterns were found in a second sample, which consisted of a larger two-wave study of 4,518 respondents. In addition, in this sample, higher other orientation mitigated the negative association between economic LMX and work effort (*b*_high_ = −0.04, *p* < 0.01 vs. *b*_low_ = −0.11, *p* < 0.001). Lastly, they found a weaker positive association between social LMX and follower OCB for followers with higher (*b*_high_ = 0.05, *p* < 0.01) than lower (*b*_low_ = 0.10, *p* < 0.01) other orientation.

Moving from follower characteristics to leader characteristics, Buch et al. (2014b) investigated the mediating role of social and economic LMX in the relationship between laissez-faire leadership and several employee outcomes. Based on two samples with 200 respondents each, they found that economic LMX fully mediated the negative relationships between laissez-faire leadership and affective commitment (standardized “effect” = −0.15, *p* < 0.01) and work effort (standardized “effect” = −0.17, *p* < 0.01), and partially mediated the relationship between laissez-faire leadership and OCB (standardized “effect” = −0.19, *p* < 0.01). These findings imply that economic LMX is an important mechanism through which laissez-faire leadership negatively relates to favorable employee outcomes.

Looking closer at leader characteristics, Kuvaas and Buch (2020) investigated the role of leader self-efficacy and leader role ambiguity on follower LMX. Based on role theory, they argued that the extent to which leaders’ experience that they meet the expectations of their leadership roles would influence the development of social and economic LMX among followers. In a sample of 109 leaders and 696 followers, they found that leader role ambiguity was negatively related to follower social LMX (γ = −0.67, *p* < 0.001) and positively related to follower economic LMX (γ = 0.52, *p* < 0.001). Additionally they found that satisfaction of the need for autonomy and relatedness mediated the relationships between both social and economic LMX and turnover intention.

Furthermore, two studies have investigated the association between social and economic LMX and follower creativity (Aleksić et al., 2017; Berg et al., 2017). Berg et al. (2017) argued that social and economic LMX would influence employee creative behavior through the followers’ willingness to take risks, depending on the follower’s emotional carrying capacity. Based on two-wave data from a sample of 147 followers, they found a marginal positive relationship between social LMX and creative behavior (*b* = 0.16, *p* = 0.06), and a non-significant relationship between economic LMX and creative behavior. Moreover, the relationship between social LMX and creative behavior was strengthened when mediated by emotional carrying capacity and moderated by willingness to take risks. Despite the fact that they only used social and economic LMX as control variables, a similar pattern was obtained by Aleksić et al. (2017) using a sample of 251 employees, revealing a positive relationship between social LMX and creativity (*r* = 0.29, *p* < 0.01), and a non-significant relationship between economic LMX and creativity.

Lastly, two studies have examined the roles of social and economic LMX in knowledge sharing processes at work (Dysvik et al., 2015; Babic et al., 2019). Dysvik et al. (2015) specifically investigated the moderating role of social and economic LMX on the relationship between follower knowledge donating and manager knowledge collecting. Based on a sample of 227 follower–leader dyads, their results revealed that there was a positive association between knowledge donating and knowledge collecting for high levels of social LMX (*b*_high_ = 0.29, *p* < 0.001) compared to low social LMX (*b*_low_ = 0.10, ns). Babic et al. (2019) investigated the role of social and economic LMX on knowledge hiding in teams. In a two-wave sample consisting of 92 teams, they found that social LMX was marginally negatively related to knowledge hiding in teams (β = −0.07, *p* = 0.093). Moreover, they found marginal support for the interaction between prosocial motivation and high levels of social LMX on the influence of knowledge hiding in the team. Both of these studies indicate that social LMX relationship is an important facilitator in knowledge sharing process at work.

In addition to the journal publications presented above, we have also included several peer-reviewed conference presentations and available book chapters in order to provide an exhausting coverage of the state of the art of social and economic LMX research.

With respect to peer-reviewed conference presentations, Buch et al. (2011) aimed to validate the scale measuring economic LMX using two independent study samples. Their findings indicate that economic LMX measure has good psychometric properties, demonstrating discriminant, convergent, and criterion-related validity (Buch et al., 2011). Moreover, they obtained negative correlations between economic and social LMX in both samples (study 1: *r* = −0.30, *p* < 0.01; study 2: *r* = −0.44, *p* < 0.01), but not strong enough to support a single continuum perspective. In relation to employee outcomes, they found that perceptions of economic LMX relationships seem to undermine affective commitment and job satisfaction, as well as increase turnover intention.

Another conference paper (De Ruiter et al., 2016) investigated the mediating roles of social and economic LMX between manager psychological contract breach and various employee outcomes. Most noteworthy is perhaps the fact that economic and social LMX differentially related to different types of outcomes. Among others, they found a negative indirect relationship from manager psychological contract breach via perceived economic LMX relationship and change related OCB, but no such significant relationship was found for social LMX. On the other hand, social LMX positively mediated the relationship between psychological contract breach and OCB directed at coworkers, but here, no significant results for the mediated influence of economic LMX were found.

Furthermore, Buch et al. (2019b) recently presented the moderating role of leader political skill, or “the ability to effectively understand others at work and to use such knowledge to influence others to act in ways that enhance one’s personal and/or organizational objectives” (Ferris et al., 2005, p. 127), on the relationship between social and economic LMX relationships and perceptions of the motivational climate. Their preliminary results indicate that perceiving social LMX positively relates to perceptions of a mastery climate, while perceiving economic LMX positively relates to perceptions of a performance climate. In support of the moderating role of leader political skill, it seems as if social LMX is especially important for the facilitation of mastery climate among less politically skilled leaders.

Despite not accounting for economic LMX, it is worth mentioning that Kopperud et al. (2018) investigated the moderating role of a leader’s psychological flexibility on the relationship between a leader’s work overload and employees’ perceived social LMX relationship. Their results suggest that when leaders have low psychological flexibility, this impairs the leaders’ ability to build social LMX relationships.

Beyond the abovementioned studies, the two-dimensional approach to LMX has also been applied in four dissertations (Buch, 2012; Alkathiri, 2016; De Ruiter, 2017; Premru, 2019) and discussed in four book chapters (Kuvaas et al., 2015; Liden et al., 2015; Buch, 2016; Buch and Kuvaas, 2016). Finally, in a chapter in the Oxford Handbook of Leader–Member Exchange (2015), Liden et al. (2015) discuss social and economic LMX as various ways of measuring LMX. In sum, dissertations, book chapters, and unpublished work indicate the same patterns as those of the published studies, while also demonstrating increased relevance across different academic sources.

## Discussion

Drawing on a number of empirical studies, the aim of our literature review was to create a comprehensive picture of the findings obtained by taking a two-dimensional approach to LMX. Our literature review suggests that social LMX demonstrates a positive relationship to a range of favorable follower outcomes. As expected, we find moderate to strong positive associations between social LMX and affective commitment; satisfaction with work family balance, employee knowledge donating, and employee resilience; and moderate negative associations with turnover intention. Positive associations are found for motivational outcomes: prosocial motivation, intrinsic motivation, need for relatedness, need for autonomy, and need for competence. Lastly, there are small to strong positive associations found between social LMX and follower performance: work effort, OCB, creativity, leader rated work performance, manager rated work effort, and manager rated OCB. These findings are as expected because of the similarity between social and high-quality LMX, and support the idea that perceiving a social LMX relationship, such as trust, as personal investment is highly beneficial for followers and their organizations.

More importantly, economic LMX is almost consistently negatively related to favorable follower outcomes. Here, we found moderate negative associations between economic LMX and affective commitment, intrinsic motivation, need for relatedness, need for autonomy, and need for competence. There are small to moderate positive associations found between economic LMX and turnover intention and extrinsic motivation. Lastly, there are small to moderate negative associations found between economic and follower performance: work effort, OCB, leader rated work performance, manager rated work effort, and manager rated OCB. This implies that experiencing an economic LMX relationship can influence the employee in several negative ways, and that there are costs to interpersonal exchanges that are more short-term, instrumental, and *quid pro quo*. In sum, the way in which social LMX and economic LMX relate differently to various outcomes across a range of different samples supports the idea of two qualitatively different relationships. As the findings illustrate, we may therefore stand at risk of losing valuable insight of the leader–follower relationship by only measuring social or high-quality LMX. By applying the two-dimensionality approach to LMX, we are arguably more able to predict important employee outcomes. In the following, we have chosen to emphasize and discuss particular findings, as we believe that they are in need of closer inspection and interpretation.

### The Two-Dimensionality of Social and Economic LMX

Beyond the link between social and economic LMX to employee outcomes, it is important to comment on the way in which they relate to each other. Generally, we found small to moderate negative associations between social and economic LMX, and three studies in which they were not significantly related at all (Aleksić et al., 2017; Babic et al., 2019; Caniëls and Hatak, 2019). This weak negative association between the two and their different relation to various employee outcomes strongly support the two-dimensional perspective of LMX. Besides, if they were opposite poles on a single continuum, the negative correlations between the two should have been much stronger.

Moreover, both empirically and theoretically, our literature review suggests that an economic LMX relationship is not the opposite of being in a social LMX relationship. Among others, Caniëls and Hatak (2019) specifically investigated the extent to which employees perceive social and economic LMX simultaneously. Their results indicate that social and economic LMX can be simultaneously present in the leader–follower relationship, manifested in the combination of equal amounts or one dominating over the other. We find additional support for the coexistence of social and economic LMX by the notion of relational ambivalence (see Ashforth et al., 2014). For instance, Methot et al. (2017) argue in favor of moving beyond the tendency to pit “negative” and “positive” workplace relationships against each other, but rather recognizing that they can be more complex (e.g., the presence of both negative and positive features at the same time). Besides, Goodwin et al. (2009) argued that the economic and instrumental behaviors associated with transactional low-quality relationships may exist over time as the relationships develop into a high-quality social exchange relationship. Therefore, an interesting avenue for future research could be to investigate how social and economic LMX relationships combine to create relational ambivalence and associated positive and/or adverse effects on different outcomes.

### Mediators Between Economic LMX and Follower Outcomes

A range of different mediators has been suggested to explain the relationship between high-quality LMX (social LMX) and subsequent follower outcomes. These mechanisms vary in their explanatory strength, but generally, the influence of mechanisms such as motivation, trust, and job satisfaction is well-established (Martin et al., 2016). Given the vast majority of research that offers insights into the underlying mechanisms between high-quality (social LMX) and follower outcomes, we focus our attention to potential mediators between economic LMX and employee outcomes.

Firstly, we believe an important avenue for future research is addressing why economic LMX is most often detrimental to follower outcomes. In alignment with suggestions made by Buch et al. (2019a), a possible explanation could be that economic exchanges operate with different exchange rules than the traditional norm of reciprocity, which is thought to be the underlying mechanism of social exchange relationships (Cropanzano and Mitchell, 2005). Rather, economic exchanges may operate by norms of competition or rivalry, where the aim of the exchange revolves around gaining more from the relationship than what you invest in it (Meeker, 1971). If so, this would serve as a potential explanation for why economic LMX relationships are associated with more negative outcomes, with the exception of some individuals that may be more inclined to respond better to more competitive norms of exchange (e.g., thriving with more exploitative exchanges). This could explain why economic LMX may be less detrimental for certain individuals (a topic that we discuss more in-depth later on).

One additional suggestion for future research could be further integration of self-determination theory (SDT) (Deci and Ryan, 1985) as an underlying explanatory mechanism. Kuvaas and Buch (2020) findings indicate that economic LMX seems to negatively influence employee outcomes partly by reducing need satisfaction. Therefore, it may be of particular interest, especially considering that social and economic LMX may serve significant, but different roles in need satisfaction, and thereby, in the process of self-determination (Kuvaas and Buch, 2020).

### Follower Characteristics and Follower LMX

A valuable recognition drawn from various studies is the potential importance of personal characteristics and dispositions in influencing how followers respond to the quality of their leader–follower relationship. By this, it is meant that individuals do not necessarily respond equally to the quality of interpersonal relationships at work (Fernet et al., 2010).

Empirical support for this is found by Buch et al. (2014a), who found that people who are highly intrinsically motivated seems to be less influenced by the benefits of a social LMX relationship, whereas people who are less intrinsically motivated have more to gain from a social LMX relationship. Additionally, Buch et al. (2019a) found that followers with high other orientation were more able to deal with the adverse influence of an economic LMX relationship, while people who were low on other orientation were the most sensitive for the negative influence of an economic LMX relationship. Similarly, we believe that a third personal factor could explain the non-significant findings in relation to economic LMX and creativity, and the marginally significant relationship found between social LMX and creativity (Aleksić et al., 2017; Berg et al., 2017). Tierney (2015) point out that the quality of the LMX relationship and its influence on creativity may matter more for some people than others. For example, Tierney (2010) found that people with a strong creative self-efficacy were not influenced by LMX, indicating that some people produce creative work regardless of the type of relationship they have with their leader (Tierney, 2015). Due to the lack of influence from social LMX and economic LMX found on followers’ willingness to take risks (Berg et al., 2017), followers’ willingness to take risks may rather function as a moderator in the relationship between LMX and creativity. This implies that economic LMX and social LMX may influence creativity differently depending on the followers’ willingness to take risks. In instances where individuals are low in willingness to take risk, social LMX may have a greater positive influence on creativity, whereas economic LMX may have a greater negative influence on creativity. Individuals who are generally not inclined to take risks will need additional support from a social LMX relationship in order to feel safe to do so. On one hand, having an economic LMX relationship may produce additional fear of failure, making them even more hesitant to take risks. On the other hand, people who generally are more willing to take risks will do so despite the quality of the LMX relationship, and therefore are also more creative.

Moreover, Caniëls and Hatak (2019) theorize in their study that narcissistic followers may respond more positively to an economic LMX relationship, as they are a “specific category of followers that require unconventional leadership treatment” (p. 21). They find that followers with narcissistic tendencies benefit from situations in which both social LMX and economic LMX are low or situations in which both social LMX and economic LMX are high (Caniëls and Hatak, 2019). This indicates that narcissism is a particular type of trait making it likely to respond differently to economic LMX relationships than the normal pattern that is often observed. In a related vein, Lee et al. (2019) found that, depending on followers’ level of psychological entitlement, he or she would value reciprocity of social exchanges differently (Lee et al., 2019). Arguably, such findings indicate that depending on an individual’s level of narcissism or other dark traits may make them respond differently or even positively to an economic LMX relationship as it may serve their expectations and worldview.

Overall, we encourage future researchers to investigate relationships between social LMX and economic LMX and outcomes *in conjunction with* potential important personal characteristics. It may be especially important to identify personal characteristics that can serve as buffers against the adverse influence of perceiving an economic LMX relationship, but also potential dark traits that enable a positive response to an economic LMX relationship.

### Leader Characteristics and Follower LMX

Limited research exists on different leader characteristics and their influence on followers’ perceived LMX relationship. However, Kuvaas and Buch (2020) found that leader role ambiguity was positively related to follower economic LMX and negatively related to follower social LMX. Since role ambiguity is often experienced as a type of strain, these leaders may simply not have the necessary resources to build social LMX relationships with their followers (Kopperud et al., 2018). Rather, it seems as if they limit their exchanges to those that adhere to the formal employment contract. Moreover, leaders may feel overwhelmed and generate withdrawal behaviors that correspond to the type of passivity that is often associated with laissez-faire leadership. This aligns well with the association found between laissez-faire leadership and economic LMX, in which economic LMX fully mediated the negative relationships between laissez-faire leadership and affective commitment and work effort, and partially mediated the negative relationship between laissez-faire leadership and OCB (Buch et al., 2014b). This means that passive leadership behaviors facilitate the perspectives of an economic LMX relationship, perhaps due to a lack of presence and exchanges that only have a more formal or transactional character. Tying together laissez-fair leadership, leader-experienced strain and economic LMX could therefore be a potential avenue for future research. Nevertheless, due to the negative association found between social LMX and role ambiguity, it seems necessary that the leader has a sufficient degree of role clarity and experienced safety within his or her role in order for the leader to be able to build social LMX relationships (Kuvaas and Buch, 2020). Overall, there are a few studies contributing to the limited knowledge regarding antecedents to economic LMX relationships. Therefore, we encourage future research to investigate factors that shape an economic LMX relationship, such as potential important leader characteristics.

### Other Areas for Future Research

We have already mentioned several avenues for future research, yet the ideas presented are by no means exhaustive, and other potential factors and possible boundary conditions need to be identified in further research. For instance, the topic of LMX differentiation has also gained increased attention in later years (Martin et al., 2018). LMX differentiation refers to how much the leader varies his or her behavior across different followers (Liden et al., 2006). In this respect, Buch (2019) recently refined the conceptualization of LMX differentiation by incorporating the distinction between social and economic LMX as part of a symposium on LMX differentiation. The aim of the symposium was to gather various articles that were thought to offer new perspective or contributions in relation to LMX differentiation.

Distinguishing between social and economic differentiation essentially implies that leaders differentiate their engagement in *both* social and economic exchanges with their followers. For instance, leaders may not differentiate social and economic exchanges equally across different followers. Rather, the leader could differentiate less in terms of economic exchanges, which means that tangible recourses may be distributed more equally, while they vary in the extent to which they engage in social exchanges with different followers. Thus, some followers may receive more supervisory support than others based on their leaders’ perception of individual needs for such support. Accordingly, distinguishing between the type of exchanges that are differentiated could have different implications for employees. For instance, perceiving that the leader engages in substantially more social exchanges with particular followers and not others may be more detrimental than perceiving variation in economic exchanges. Also, and as hypothesized by Buch (2019) at the recent LMX symposium, these kinds of differentiation may be “good” or “bad” for employee outcomes, depending on, for instance, whether the followers prefer short-term oriented economic exchanges (often of tangible resources, and characterized by little personal involvement) or more long-term, diffuse social exchange relationships.

Although more research is clearly needed, Buch (2019) preliminary “work-in progress” results presented at last year’s LMX differentiation symposium (EAWOP) provided an early empirical indication that more politically skilled leaders differentiated less on the economic exchange dimension. Tentatively, leaders may have done so to avoid or to reduce feelings of inequality or injustice because, theoretically, more tangible economic exchanges should be more salient to others, and leads to jealousy and envy to a greater extent. Buch’s (2019) preliminary results also suggested that economic LMX and social LMX differentiations were barely correlated, and that a higher leader span of control (the more followers each leader had to supervise) was related to more differentiations on the social exchange (but not the economic exchange) dimension of LMX. Accordingly, one could hypothesize that, with larger groups, leaders tend to differentiate more on the social exchange dimension where they choose their reciprocal relationships, and differentiate between/selected exchange partners in a trusting and long-term oriented way. As a whole, this refinement of LMX differentiation is likely to have implications for how leaders differentiate between their use of social and economic exchanges in their LMX relations. For example, the more differentiated the distribution of tangible, economic exchanges, the less they could be considered “negative,” given the conditions such as a politically skilled leader demonstrating individualized consideration and treating individuals differently according to their perceived and actual needs.

As such, applying a similar two-dimensional logic to the LMX differentiation literature could be an interesting avenue for future research. We therefore encourage future research to investigate these matters further and to consider the inclusion of potential moderators such as leader political skill (e.g., Buch, 2019), justice (e.g., Yu et al., 2018), and leadership behaviors such as transformational leadership (e.g., Gottfredson and Aguinis, 2017).

Moreover, a fruitful avenue for future research could be considering the issue of LMX (dis)agreement when applying the two-dimensional approach to LMX. A great amount of LMX research has only captured the perspective from one member of the dyad, since it has been assumed that a leader and a follower will evaluate the quality of their relationship similarly (Gooty and Yammarino, 2016). However, it is increasingly recognized that agreement in terms of how the leader and the follower evaluate the quality of their relationship is, at the general level, often only moderate at best (Gerstner and Day, 1997; Sin et al., 2009). Distinguishing between social and economic LMX as two different types of relationships will necessarily have implications for how we understand the phenomenon of (dis)agreement that deviates from how it is traditionally understood. Discrepancy found on traditional measures of LMX may only reflect divergent perceptions regarding the absence or presence of socioemotional qualities within the leader–follower relationship. This is not sufficient to conclude that traditional LMX disagreement actually indicates divergent perceptions regarding the more economic or transactional qualities within the leader–follower relationship. As such, measuring perceptions of social and economic LMX from both sides of the dyad allows for nuancing the LMX (dis)agreement phenomenon and could potentially contribute to an enhanced understanding of its relation to employee outcomes.

Although some studies have applied leader-rated performance measures, future research should also consider applying objective measures in relation to the influence of social and economic LMX. Our literature review provides empirical support for the importance of measuring economic LMX in addition to social or high-quality LMX, but adding objective outcome measures could potentially increase our confidence even more. Overall, we believe that our suggested potential areas of future research may enhance our understanding of the leader–follower relationship even further.

## Conclusion

As LMX has gained increased research attention, the field has also been criticized for a lack of fit between theory and how LMX has been measured. Still, such criticism provides opportunities for future advancement. By providing a literature review of social and economic LMX, we aim to contribute to constructive development of the field by strengthening the tie between the LMX theory and the SET. We also aim to encourage researchers to develop the two-dimensional perspective even further in future research.

## Author Contributions

IA wrote most of the first draft of the manuscript. RB contributed by writing parts of the manuscript. All authors provided critical feedback and helped shape the manuscript.

## Conflict of Interest

The authors declare that the research was conducted in the absence of any commercial or financial relationships that could be construed as a potential conflict of interest.
